# The Impact of Antiretroviral Therapy on Mortality in HIV Positive People during Tuberculosis Treatment: A Systematic Review and Meta-Analysis

**DOI:** 10.1371/journal.pone.0112017

**Published:** 2014-11-12

**Authors:** Anna Odone, Silvia Amadasi, Richard G. White, Theodore Cohen, Alison D. Grant, Rein M. G. J. Houben

**Affiliations:** 1 TB Modelling Group, Centre for the Mathematical Modelling of Infectious Diseases, London School of Hygiene and Tropical Medicine, London, United Kingdom; 2 Department of Global Health and Social Medicine, Harvard Medical School, Boston, Massachusetts, United States of America; 3 University of Parma, School of Medicine, Parma, Italy; 4 University Division of Infectious and Tropical Diseases, University of Brescia and Spedali Civili General Hospital, Brescia, Italy; 5 TB Centre, London School of Hygiene and Tropical Medicine, London, United Kingdom; 6 Center for Communicable Disease Dynamics, Department of Epidemiology, Harvard School of Public Health, Boston, Massachusetts, United States of America; 7 Division of Global Health Equity, Brigham and Women's Hospital, Boston, Massachusetts, United States of America; 8 Department of Clinical Research, London School of Hygiene and Tropical Medicine, London, United Kingdom; London School of Hygiene and Tropical Medicine, United Kingdom

## Abstract

**Objective:**

To quantify the impact of antiretroviral therapy (ART) on mortality in HIV-positive people during tuberculosis (TB) treatment.

**Design:**

We conducted a systematic literature review and meta-analysis. Studies published from 1996 through February 15, 2013, were identified by searching electronic resources (Pubmed and Embase) and conference books, manual searches of references, and expert consultation. Pooled estimates for the outcome of interest were acquired using random effects meta-analysis.

**Subjects:**

The study population included individuals receiving ART before or during TB treatment.

**Main Outcome Measures:**

Main outcome measures were: (i) TB-case fatality ratio (CFR), defined as the proportion of individuals dying during TB treatment and, if mortality in HIV-positive people not on ART was also reported, (ii) the relative risk of death during TB treatment by ART status.

**Results:**

Twenty-one studies were included in the systematic review. Random effects pooled meta-analysis estimated the CFR between 8% and 14% (pooled estimate 11%). Among HIV-positive TB cases, those receiving ART had a reduction in mortality during TB treatment of between 44% and 71% (RR = 0.42, 95%CI: 0.29–0.56).

**Conclusion:**

Starting ART before or during TB therapy reduces the risk of death during TB treatment by around three-fifths in clinical settings. National programmes should continue to expand coverage of ART for HIV positive in order to control the dual epidemic.

## Introduction

Infection with HIV dramatically increases individuals' risk of developing active tuberculosis (TB) disease, as well as the risk of death during TB treatment [1]. While 13% of all people with TB are estimated to be HIV positive, they account for approximately a quarter of TB deaths [2]. The African region, where 250,000 deaths occurred among HIV-positive TB cases in 2012, accounts for 75% of HIV-positive TB cases [2].

Globally, in 2012, 46% of notified TB cases had a documented HIV test result and the coverage of Antiretroviral therapy (ART) among TB patients known to be HIV-positive was estimated to be 57% [2]. ART reduces the impact of HIV on incident TB, as illustrated by Suthar *et al.* who showed that the risk of TB is reduced by 65% [3]. Also, results from various trials and observational studies have consistently shown a benefit of ART on TB outcomes, and indicated that ART should be initiated as early as possible in the course of a TB episode [4], [5], [6], [7], [8], [9], [10], [11]. However, to date, there has been no systematic overview of the magnitude of benefit of ART on TB mortality. A recent systemic review by Straetemans *et al.* on TB mortality stratified subjects by HIV status but did not explore the effect of ART [12].

TB mortality in HIV-positive people is a key parameter to describe current state and progress in TB care and control [2]. Ideally, TB mortality is estimated through direct measurements using vital registration systems or mortality surveys, but these are often unavailable in resource limited settings, where TB incidence is highest [2]. Alternatively, TB mortality can be estimated indirectly by calculating the case fatality ratio (CFR), which is defined as the proportion of people with incident TB that die as a result of this episode of disease [13]. TB-CFR has been defined by Maher *et al.* as the proportion of TB cases that die within a specified time [14]. In particular, Mukadi *et al.* defined TB-CFR as the proportion of tuberculosis patients that die during TB treatment [15], which is the definition applied by Straetemans *et al*. in their review [12].

In this paper we systematically review the literature to estimate the mortality during TB treatment among HIV-positive TB patients receiving ART as well as its value relative to HIV-positive TB patients not receiving ART.

## Methods

The review's methods were defined in advance following the Prepared Items for Systematic Reviews and Meta-Analysis (PRISMA) guidelines [16].

### Criteria for considering studies

#### Study population

We included studies that described mortality among HIV positive patients receiving TB treatment who initiated combination ART before or during TB treatment. All sex and age groups were included.

Our focus was on estimating the effect of ART as it applied to TB patients from the general population, and we excluded cohorts that were limited to recurrent, extra-pulmonary or known drug-resistant TB patients only. We also excluded studies focusing on populations of patients not representative of the general population such as miners [17], prisoners [18], healthcare workers [19] or injecting drug users [20].

#### Study design and sample size

Eligible study designs included clinical trials, prospective cohort, retrospective cohort and case-control studies. Literature reviews were screened to retrieve relevant primary data. Studies which reported mortality results on fewer than 50 TB patients receiving ART were also excluded.

#### Outcome measures

We focused on deaths occurring *during* TB treatment, the main operational parameter used in the field by most TB control programmes. This outcome has the benefit of a clearly defined at-risk period which facilitates between-study comparisons as well as programmme monitoring and evaluation. Papers were therefore excluded if they did not report mortality within six to eight months after start of TB treatment or otherwise restrict patient follow-up to the end of treatment.

We considered two primary outcome measures. Firstly, the TB-CFR defined as percentage of deaths among the study population occurring during TB treatment. Secondly, if the paper also reported TB mortality in HIV-positive individuals not on ART, we recorded the estimated relative risk (odds ratio (OR), relative risk (RR) or hazard ratio (HR)) of death during TB treatment by ART status.

### Search methods for identification of studies

Studies were identified by searching the electronic databases Medline and Embase. The strategy was first developed in Medline and then adapted for use in the other databases (Appendix A). Studies published in English from 1996 (when combination ART first became available) through July2014 were included. In addition, the abstract books of the world conferences on lung health of the International Union against Tuberculosis and Lung Diseases (IUATLD) were manually scanned for the period 2004–2013 and further studies were retrieved from reference lists of relevant articles and consultation with experts in the field.

### Data collection and analysis

#### Data extraction

Identified studies were independently reviewed for eligibility by two authors in a two-step process; a first screen was performed based on title and abstract while full texts were retrieved for the second screen. At both stages disagreements between reviewers were resolved by discussion.

Data were extracted by one author supervised by a second author using a standardised data extraction spreadsheet. The data extraction spreadsheet was piloted on 10 randomly selected papers and modified accordingly. Data extraction included: authors' names, year and country of publication, study design, study setting, study period, age of study participants, information on TB type, TB diagnosis and drug resistance, information on time of ART initiation, follow-up time, information on analysis performed and outcomes of interest. We defined TB treatment to be standardized (following the definition used by Straetemans [12]) if it was described to be: i) in line with WHO recommendations, ii) following national or governmental guidelines, iii) direct observed treatment strategy (DOTS) or iv) defined as ‘standard’ [12].

#### Analysis

We performed descriptive analysis to report the characteristics of the included studies. Midpoint values for the age of the included cohorts was based on the reported mean or median and were pooled as weighted averages. With regard to the pre-specified outcomes, we would expect variability between studies, e.g. based on average CD4 count in the study population or general quality of local health systems. We therefore applied random effects analyses to acquire an estimate of the average effect of ART on TB mortality, rather than assuming a single true value in a fixed effects approach [21]. With regard to the pre-specified outcomes, heterogeneity was assessed using the I^2^ statistic and visual inspection of forest plots [21]. Depending on data availability, we planned to conduct sub-group analyses (where relevant and possible) by WHO region, age-group, time interval between TB treatment start and ART start, laboratory-confirmed TB diagnosis and median CD4 count at start of study. If unadjusted and adjusted outcomes were available, we recorded the adjusted estimate to reduce the risk of confounding.

#### Quality assessment

The same two authors who performed data extraction independently assessed the quality of the included studies using the Newcastle-Ottawa Assessment Scale (NOS) [22]. Level of quality was not set as a criterion for exclusion. Disagreements by reviewers were resolved by consensus.

## Results

### Identified studies

**Figure 1:**
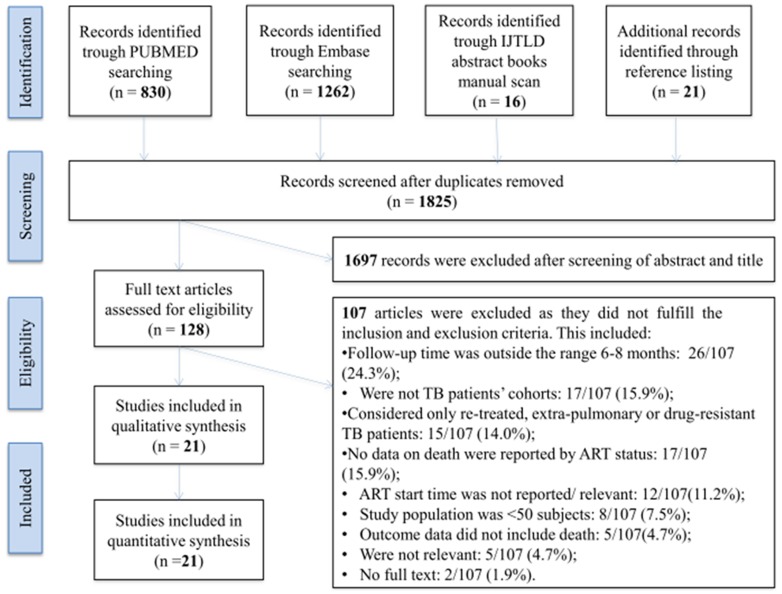
PRISMA flow diagram of papers selected.

We identified 2,129 records by searching the selected databases and listing references of relevant articles. After removing duplicates, 1,825 articles were retrieved. Papers were screened and selected as illustrated in Figure 1, resulting in 21 studies that were included in the systematic review and meta-analysis.

### Characteristics of included studies

The characteristics of the included studies are reported in Table 1. The majority of the studies were conducted in the African (n = 11, 52%) [14], [23], [24], [25], [26], [27], [28], [29], [30], [31], [32] and South-East Asian (n = 7, 33%) [33], [34], [35], [36], [37], [38], [39] regions; two studies were conducted in South America [40], [41] and one in Europe [42]. Four out of seven studies in the South-East Asian region were conducted in Thailand [33], [35], [37], [38] while one used data from eighteen sites in the Asia-Pacific region [39]. Both South American studies were conducted in Brazil.

**Table 1:** Characteristics of included studies.

Reference	Country	WHO Region	Study Design	Study Period	Patient Source	Population		Age b (Years)	PTB (%)	New TB Cases (%)	MDR-TB (%)	Standardized TB Treatment	Follow-Up Period	ART Start Time	
						Main Study	For Review (%)							Before TB (%)	During TB Treatment ^C^ (Midpoint (Range))
Agodokpessi 2012 [23]	Benin	AFRO	Cohort (Ret)	Jan–Dec 2009	1 Urban Hospital	259	85 (33)^A^	36 (15–72)$	88$	Na	Na	Yes	TB Treatment	0	Na
Akksilp 2007 [33]	Thailand	SEARO	Cohort (Pros)	Feb2003–Jan2004	25 Health Clinics	329	75 (23)	32 (1–68)$	69$	93$	1$	Yes	TB Treatment	40	93 (0–170)
Dean 2002 [42]	UK	EURO	Cohort (Ret)	Jan1996–Jun1999	12 Urban Hospitals	188	85 (45)	34 (21–70) $	51$	Na	7$	Not All	TB Treatment	18	60 (0–14)
Dos Santos 2013 [40]	Brazil	AMRO	Cohort (Ret)	Jan1995–Dec 2003	2 Urban Hospitals	347	191 (55)	Na	63$	Na	0	Yes	TB Treatment	0	Na
Ferrousier 2013 [28]	Benin	AFRO	Cohort (Ret)	Jan 2006–Jan 2008	20 Health Clinics	1255	462 (37)	83% aged 16–45$	91$	67$	0.1$	Na	TB Treatment	44	60 (TB treatment intensive phase)
Gandhi 2012 [14]	South Africa	AFRO	Cohort (Pros)	Oct 2003–Jan 2006	1 Rural Hospital	119	119 (100)	34 (±7)	Na	Na	Na	Yes	TB Treatment	0	67 (60–83)
Henegar 2012 f [24]	DRC	AFRO	Cohort (Ret)	Jan 2006–May 2007	14 Health Clinics	933	129 (14)	38 (±10)$	66$	80$	Na	Yes	TB Treatment	36	Na
Kaplan 2013 [29]	South Africa	AFRO	Cohort (Ret)	Jan 009–Dec 2011	100 Health Clinics	77499	21851 (28)	34 (28–40) $	76$	69$	0	Yes	TB Treatment	24	Na
Kayigamba 2013 [30]	South Africa	AFRO	Cohort (Ret)	Jan–Apr2007	48 Health Clinics	581	110 (19)	31 (25–41) $	72	100	Na	Yes	TB Treatment	66	179^1^
Kendon 2012 [25]	South Africa	AFRO	Cohort (Ret)	Jan 2008–Dec 2010	1 Urban Hospital	468	388 (83)	35 (31–42)	Na	Na	Na	Na	6 Months From ART Start	0	(0–56)
Nansera 2012 [26]	Uganda	AFRO	Cohort (Pros)	Feb 2007–Mar 2010	1 Urban Hospital	386	228 (59)	33 (18–69)$	83$	90$	Na	Yes	TB Treatment	30	49 (4–18)
Raizada 2009 [34]	India	SEARO	Cohort (Ret)	Mar 2007–Aug 2007	154 Health Clinics	734	380 (52)	34 (8–89) $	75$	87$	Na	Yes	TB Treatment	35	Na
Sanguanwongse 2008 [35]	Thailand	SEARO	Cohort (Pros)	Oct 2004–Mar 2006	1 Urban Hospital + Several Healthcare Clinics	1269	626 (49)	34 (1–71)	48	100	1$	Not All	TB Treatment	0	Na
Schmaltz 2009 [41]	Brazil	AMRO	Cohort (Pros)	Apr 2000–Jul 2005	1 Urban Hospital	106	83 (78)	Na	49$	Na	7$	Yes	TB Treatment	41	43 (28–74)
Sileshi 2013 [31]	Ethiopia	AFRO	Cohort (Ret)	Apr 2009–Jan 2012	1 Urban Hospital + 3 Health Clinics	422	272 (64)	30 (27–37.5)	44$	78	Na	Na	TB Treatment	Na	Na
Sinha 2012 [36]	India	SEARO	RCT	May 2006–Mar 2011	1 Urban Hospital	150	150 (100)	35 (±8)	23	Na	0	Yes	6 months/TB Treatment Completion	0	41 (14–84)
Tansuphasawadikul 2007 [37]	Thailand	SEARO	Cohort (Ret)	Jan 2004–Jun 2005	1 Urban Hospital	101	82 (81)	33 (20–58)	19	86	6	Na	TB Treatment	0	68 (0–381)
Tweya 2013 [32]	Malawi	AFRO	Cohort (Ret)	Jan 2008–Dec 2010	1 Urban Hospital	2478	492 (20)	31 (26–38) $	100	100	Na	Yes	TB Treatment	0	60
Varma 2009 [38]	Thailand	SEARO	Cohort (Pros)	May 2005–Sept 2006	1 Urban Hospital + 32 Health Clinics	667	273 (41)	34 (18–77)$	58$	100	2$	Not All	TB Treatment	27	62 (0–386)
Zachariah 2007 [27]	Malawi	AFRO	Cohort (Ret)	Jan–Dec 2004	1 Rural Hospital	983	180 (18)	32 (2–74)	79	100	Na	Yes	TB Treatment	0	88 (66–125)
Zhao 2014 [39]	Asia	SEARO WPRO	Cohort (Pros)	Sep 2003–May 2004	18 sites	768	429 (56)	34 (29–39) $	42$	Na	Na	Na	TB Treatment	191	42 (17–64)

RCT: Randomized Controlled Trial; Pros: prospective; Ret: retrospective PTB: pulmonary tuberculosis; DS: drug-sensitive; DR: drug-resistant; Na: not available; IQR: interquartile range.

$ Values refer to the total population of main study (in absence of detailed data available on TB cases receiving ART).

a Total study population included also 827 HIV-negative subjects. Data in the table refer to the HIV positive subset.

b reported estimated midpoint + range or SD; ^c^ (in days), relative to TB treatment start/TB diagnosis date, range either complete range or IQR depending on data availability.

c refers to subjects included in the meta-analysis. Detailed information on CD4 count for the whole study population for each study is reported in Table S1.

d1 subjects already on ART by the start of TB treatment,

d2 subjects that started ART within 90 days from TB treatment start.

e range.

1 21% (n = 37) of them started within 3 months of TB treatment start.

More than half of included studies (n = 13, 62%) described a retrospective cohort [23], [24], [25], [27], [34], [37], [40], [42], seven (33.3%) described prospective cohort studies [14], [26], [33], [35], [38], [41] and one was a clinical trial [36]. We excluded seven papers [5], [6], [7], [8], [9], [43], [44] reporting results from large trials that focused on time of ART initiation during TB treatment as TB mortality data was only reported at time points that lay beyond our pre-specified ‘during treatment’ period [6], [7], [43], [44], the outcome was death combined with AIDS-defining illness and mortality data could not be extrapolated [7] or focused only on tuberculous meningitis [9]. Study enrollment periods ranged from 4 months [30] to 8 years [40]. The majority of the studies were conducted predominantly in urban settings (n = 14, 66.7%) [12], [23], [24], [25], [26], [29], [30], [31], [32], [36], [40], [41], [42]. Table 2 shows the levels of quality assigned to each study. We determined that the majority of the studies were susceptible to mild cohort selection bias because of their retrospective study design [23], [24], [25], [27], [34], [37], [40], [42]. We did not assess ten eligible studies for comparability as they were descriptive studies not reporting effect estimates [14], [23], [25], [27], [28], [31], [36], [37], [39], [42]. Among the rest, we judged that five had moderate comparability, because confounding factors were not fully adjusted or they were not specified [24], [29], [30], [34], [35]. For most studies, the assessment of outcomes was done by medical record review which we regarded as susceptible to mild outcome bias (Table 2).

**Table 2:** Source of data, method of death ascertainment or confirmation and quality assessment of the included studies.

Reference	Source of data and method of data collection	Method of death ascertainment/confirmation	The Newcastle-Ottawa Scale		
			Selection	Comparability	Outcome
Agodokpessi 2012 [23]	Data were extracted from medical records	Na	***		**
Akksilp 2007 [33]	Surveillance and monitoring data from public health program	Na	***	**	**
Dean 2002 [42]	Data were extracted from medical records	Na	**		**
Dos Santos 2013 [40]	Data were extracted from medical records by trained health care workers	Na	***	**	**
Ferroussier 2013 [28]	Data were extracted from TB registers	Na	***		**
Gandhi 2012 [14]	Na	Na	**		**
Henegar 2012 [24]	Na (we assume Data were extracted from medical records)	Na	**		*
Kaplan 2013[29]	Data were extracted from the national electronic TB register	Na	***	*	**
Kayigamba 2013 [30]	Data were extracted from TB registers and TB treatment charts	Na	**		**
Kendon 2012 [25]	Data were extracted from medical records	Na	**		**
Nansera 2012 [26]	Na	Na	**	**	*
Raizada 2009 [34]	Data were extracted from medical records	Na	***	*	**
Sanguanwongse 2008 [35]	Data were extracted from medical records	Na	***	*	**
Schmaltz 2009 [41]	Data were extracted from medical records	Na	**	*	**
Sileshi 2013 [31]	Data were extracted from medical records (Pre-ART registers, lab requests, follow-up forms, anti TB record forms, ART intake forms, and patient cards).	The patients' date of death was extracted from TB registration log books	*		**
Sinha 2012 [36]	Clinical and laboratory data actively collected and reported on an *ad hoc* electronic data base.	Na	-^a^	-^a^	-^a^
Tansuphasawadikul 2007 [37]	Data were extracted from medical records	Na	*		**
Tweya 2013 [32]	Data were extracted from TB registers and TB treatment cards. Deaths were ascertained mainly through active follow-up.	Na	***	**	**
Varma 2009 [38]	Data were extracted from medical records and the Thailand TB Active Surveillance Network.	To determine if patients died after defaulting notification data were linked to the Thai government's vital status registry.	***	**	**
Zachariah 2007 [27]	Data were extracted from TB registers (counselling registers, district TB registers, TB patient cards, ART Patient Master Cards and ART Registers)	Na	**		**
Zhao 2014 [39]	TREAT Asia HIV Observational Database (TAHOD)	Death was confirmed by local medical staff and reported using standardized Cause of Death (CoDe) forms	**		**

-a Newcastle-Ottawa Quality assessment scale not applicable (study design: randomized trial, higher level of evidence as compared to observational studies [60].

### Characteristics of the study populations

86% (n = 18) studies reported on source of data and method of data collection; of these, data were mostly collected from medical records (n = 10, 56%) and TB registers (n = 5, 28%). Three studies explicitly reported on how death was ascertained or confirmed (Table 2) [38], [39], [45].

For most studies (n = 19, 90.5%) only a fraction of the total patient population included TB cases that started ART before or during TB treatment. For example, in Agodokpessi et al., of the total patient population, 259/1086 (24%) were HIV positive and of these 259, 85 (33%) initiated ART before or during TB treatment [23]. On average, 50% of the studies' study population (range: 12–100%) fitted our criteria (Table 1). The number of included patients per study ranged from 75 [33] to 21,851 [29], with a median of 191 subjects. Studies included predominantly young adults (age midpoint: 34. years, SD: ±1.9).

All but two [14], [25] studies reported on TB type: among them, the percentage of individuals with pulmonary TB ranged between 19.4% and 100% (median: 66%). Where reported, most subjects were new TB cases (median percentage: 90%, range: 67%–100%). Drug susceptibility profiles were reported by nine studies. Three studies only included drug-sensitive TB cases and in the remaining studies, the proportion with multi-drug resistance was low and ranged from 0.1%–7% (median: 1.5%; included in the review). Three studies reported that some TB patients did not receive standardized therapy [35], [38], [42]. Details on TB treatment regimens are available in Table S3. Descriptions of TB type, TB category and drug susceptibility in the majority of studies referred to the original studies' entire cohorts and not to the subsets of the study population selected for this analysis.

Eleven studies reported that a proportion of their patients initiated ART before TB treatment (between 18 and 66%, Table 1), but the actual ART start dates were not reported [24], [26], [28], [29], [30], [33], [34], [38], [39], [41], [42]. Where all patients were initiated on ART during TB treatment, the median start times occurred in month 2 or 3 after starting TB treatment [14], [25], [27], [32], [36], [37]. The variability in reporting between studies limited our ability to examine the mortality impact of different ART initiation times with respect to the start of TB treatment.

Data on CD4 count were very heterogeneous and incomplete and did not allow quantitative assessment of effect modification (Table S2). In six (29%) studies, data on CD4 count were not reported at all [24], [27], [30], [32], [34], [40]; in four (19%) studies they were not available for the subgroup of the study population included in the meta-analysis [14], [33], [38], [41]; and in five (24%) studies the percentage of the study population included in the meta-analysis for which some measures of CD4 count were available did not exceed 55% [23], [26], [28], [37], [42]. Median CD4 count was available for >98% of subjects included in the meta-analysis in four (19%) studies [31], [35], [36], [39] and ranged from 48 to 152 cells/mm^3^.

Follow-up time corresponded to TB treatment duration in 18 (75%) studies. In Sinha *et al*., follow-up time was set at six months after TB treatment start or at TB treatment completion, and in Kendon *et al*., it was set at six months from ART start (ART was started within 56 days from TB treatment start) [36].

One study reported outcomes which were limited to bacteriologically-confirmed (positive sputum smear or culture result) TB cases [27], [35] and one study included only culture-confirmed TB cases [35].

### Outcome measures

**Figure 2:**
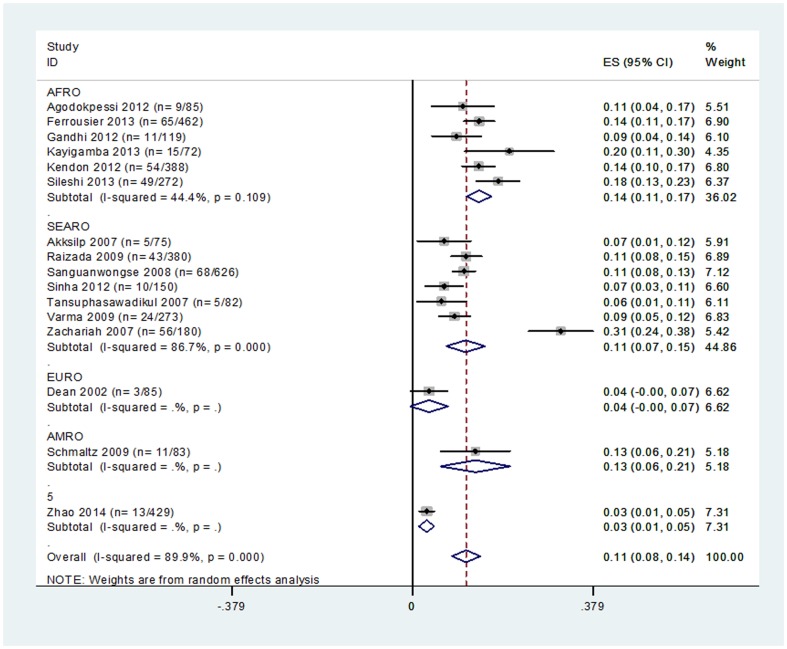
Forest plot of 16 studies reporting TB-CFR for HIV positive patients receiving ART (by Region).

Sixteen studies reported data on CFR (Table 3) [14], [23], [25], [27], [28], [30], [31], [33], [34], [35], [36], [37], [38], [39], [41], [42]. Meta-analysis of the CFR showed high heterogeneity (I-squared  = 89.9%, p<0.001, Figure 2). The random-effects analysis suggested the CFR lay between 8% and 14% (point value  = 11%; Figure 2). The pooled CFR appeared slightly higher in the African region (11%–17%), and lower in the South-East Asian (7%–15%) region. The CFR was 4% (95%CI: 0%–7%) and 13% (95%CI: 6%–21%) in single studies set in the UK and Brazil respectively (Figure 2). Sanguanwongse *et al*. considered a subgroup of patients with very low CD4 counts at the start of TB treatment (CD4 <10 cells/mm3), which experienced a CFR of 21% (95%CI: 1%–32%) [35]. When restricted to the four studies where all patients were initiated on ART after starting TB treatment, the CFR was 12% (95% CI 8%–17%).

**Table 3:** Outcomes considered in the included studies.

Reference	Subgroups	Sample size	N. Deaths	TB-CFR (%)	Effect estimate type	Effect estimate value (95% CI)	Univariable/Multivariable (adjusted for)
Agodokpessi 2012 [23]		85	9	11			
Akksilp 2007 [33]		75	5	7	RR	0.2 (0.1–0.4)	Multivariable (CD4, smear status, co-trimoxazole use, treatment facility)
Dean 2002 [42]		85	3	4*			
Dos Santos 2013 [40]		191			HR	0.1 (0.03–0.29)	Multivariable (age, sex, marital status and total lymphocyte count)
Ferroussier 2013 [28]		462	65	14			
Gandhi 2012 [14]		119	11	9			
Henegar 2012 [24]		129			IRR	0.63 (0.36–1.10)	Univariable
Kaplan 2013 [29]	On ART at start of TB	21851			OR	0.53 (0.46–0.60)	Multivariable (na)
	Started ART during TB				OR	0.42 (0.39–0.47)	Multivariable (na)
Kayigamba 2013 [30]		72	15	21	OR	1.43 (1.28–1.61)	Univariable
Kendon 2012 [25]		388	54	14			
Nansera 2012 [26]		228			HR	0.13 (0.07–0.25)$	Multivariable (sex and disease category)
Raizada 2009 [34]		380	43	11	HR	0.41 (0.28–0.6)	Multivariable^a^
Sanguanwongse 2008 [35]	All	626	68	11	RR	0.18 (0.13–0.25)	Multivariable^b^
	Only bact. confirmed	583£			RR	0.15 (0.09–0.24)	Multivariable^b^
	CD4 <10 cell/mm^3^	56	12	21	RR	0.26 (0.16–0.44)	Multivariable^b^
Schmaltz 2009 [41]		83	11	13	HR	0.55 (0.52–0.59)*	Multivariable^b^
Sileshi 2013 [31]		272	49	18			
Sinha 2012 [36]		150	10	7			
Tansuphasawadikul 2007 [37]		82	5	6			
Tweya 2013 [32]		492			OR	0.46 (0.26–0.83)	Multivariable (sex, age, HIV status, registration year)
Varma 2009 [38]	All	273	24	9	HR	0.16 (0.07–0.36)	Multivariable (CD4, TB severity)
	Only bact. confirmed	na			HR	0.06 (0.02–0.23)	Multivariable (CD4, TB severity)
Zachariah 2007 [27]		180	56∧	31			
Zhao 2014 [39]		429	13	3			

*Calculated with the available data.

$ Assumed to be a coding error in the original article (reciprocal HR reported).

∧ Deaths occurred during TB treatment's initial phase.

£ Assuming all 110 culture positive patients were not included in 473 smear positive patients.

a Variables to include in the model were chosen based on the literature.

b Variables to include in the model were chosen for inclusion in the multivariate analyses based on one or more of the following: p<0.20 in bivariate analysis, biologic plausibility, or previously published evidence.

**Figure 3:**
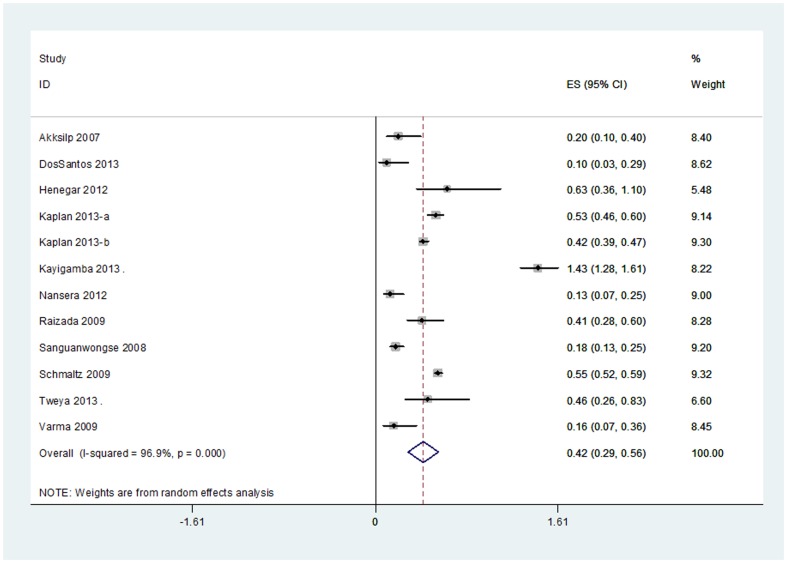
Forest plot studies reporting the relative risk of death during TB treatment by ART status.

Eleven out of 21 studies reported data on estimated relative risk of mortality comparing people taking vs. not taking ART [24], [26], [29], [30], [32], [33], [34], [35], [38], [40], [41]. Adjusted relative risks were available for all but one study [26]. A random effects meta-analysis showed that the relative risk of death during TB treatment by ART status was 0.42 with a 95%CI: 0.29–0.56; (I-squared  = 96.9%, p<0.001, Figure 3), which corresponds to a 44% to 71% reduction in mortality. When we restricted the analysis to studies considering only patients with smear positive TB (n = 2) the random effects relative risk was 0.11 (95%CI: 0.03–0.20) [35], [38].

## Discussion

We estimate that mortality during TB treatment in HIV-positive individuals receiving ART under routine programmatic conditions lies between 8% and 14% and that ART reduces the mortality during TB treatment for HIV-positive TB cases by between 44 to 71%.

This is the first systematic assessment to quantify the impact of ART on TB mortality during TB treatment. In addition, we report not only pooled absolute mortality figures as done elsewhere [12] but also estimate the relative effect of ART on mortality, which has the advantage of being a less setting-specific and a more easily interpretable and generalizable parameter.

Our focus on deaths *during* TB treatment allowed us to make optimal use of the limited data, while providing a clear conceptual parameter that is widely used and reported by National TB Programmes, and which allows comparison between studies and other reviews. Mortality estimates over longer follow-up periods have been reported in recent trials [6], [7], [8], [43], but are not usually reported in surveillance systems, operational research and clinical settings. Previous reviews and reports have discussed the difficulties in trying to determine which deaths are directly attributable to TB, especially in low-resource settings [2], [12]. Different studies classified TB as either the primary or a contributory cause of death [12], [46]. In addition, according to the 10th revision of the International classification of diseases (ICD-10), deaths in HIV-positive TB patients are classified as HIV deaths [1]. In light of these considerations, we systematically assessed the effect of ART on death *during* TB treatment rather than deaths *due* to TB.

While the exclusion of several large RCTs reduced the number of studies and patients included, it was necessary to ensure between-study comparability regarding the time at risk of death. When reported, we note that these RCTs found relatively comparable TB-CFRs to our review [7]. We note that in the CAMELIA trial, the CFR was substantially higher than our estimate (18% and 27% for those initiating ART two or eight weeks into TB treatment), but this is likely to be due to the very low baseline CD4 count of patients included in that study (median  = 25 cells/mm^3^) and the longer follow-up time (median  = 25 months) [6]. As expected, other observational studies that considered follow-up periods greater than 3.5 years also find higher absolute mortality estimates [47].

The estimated range for CFR estimate of 8–14% in TB patients receiving ART is also below than the 19% point value reported by Straetemans *et al*. amongst all HIV-positive TB cases, which included some individuals receiving ART. This is consistent with expected effect of ART to reduce TB mortality [12], [27].

Sub-group analyses by geographical region suggested that the CFR was higher in the African region (14%, estimates based on 7 studies) and lower in the one study from a Western European setting (4%, in the UK), which mirror global TB mortality patterns [2]. The reported differences in CFR between regions might also be attributable to differences in immunosuppression at the time of clinical presentation which could affect the estimated benefits of ART. The protective effect of ART appeared stronger in people with smear-positive TB, reflecting the better treatment outcomes in this population across HIV/ART groups compared to patients without smear or culture confirmation. This could be due to some people who do not genuinely have TB being erroneously included in the smear negative group. However, given the small number of studies with bacteriological confirmation (n = 2), this result should be interpreted with caution.

A limitation of our work is that – due to a lack of data - we could not explore the effect of ART by CD4 count. The lack of data is due to the fact that the majority of the included studies reported data from TB registers, and surveillance systems in high HIV-TB burden and resource-limited settings where CD4 measurements are often unavailable [48]. Recent data from large RCTs have provided solid evidence that ART reduces mortality across a wide range of CD4 counts [5], [6], [7]. In addition, the 2013 WHO guidelines for antiretroviral therapy provide the evidence-based recommendation that in subjects with active tuberculosis ART should be initiated as soon as possible, irrespective of CD4 count [49]. However, we found that on average patients were started two to three months post TB treatment initiation, suggesting this may not have been policy or practice in the majority of observational studies included. If ART were provided to all HIV positive TB patients as per current guidelines, we would expect the CFR to decrease [8], [43], and to see an even greater reduction in relative mortality. The question of when to start ART in HIV-positive TB patients has been addressed by the above-cited RCTs [5], [6], [7], [8], [50] and - based on their findings - HIV and TB guidelines recommend that among HIV-positive TB patients with CD4 less than 50 cells/mm^3^, ART should be initiated within 2 weeks from TB treatment start and if CD4 above 50 cell/mm^3^, within 8 weeks [51], [52], [53], [54], [55]. Nonetheless, the issue remains controversial as results from a recent trial reported no difference in mortality between early and delayed ART for HIV-positive TB patients with CD4 counts of 220 cells/mm^3^ or more with authors arguing WHO guidelines should be updated accordingly [44] while, on the other hand, some researchers question the need of investing resources in other randomized, controlled trials on the same topic [56].

Nearly all data came from observational studies, which are more vulnerable to bias. For example, sicker patients might be more likely to be selected to start ART, thus introducing selection bias that would under-estimate the effect of ART. However, observational studies may better represent the patient population and clinical care provided in most settings. The majority of the studies considered single hospitals, local or district-level data and this might limit the ability to generalize results to national or regional level. Patients seen at referral hospitals may be sicker and may experience higher mortality, while at the same time, those treated in urban centres might have lower mortality than those treated in rural settings. Last but not least, most studies relied on TB notification registers and medical records whose degree of completeness and quality of reporting might be different in different settings, potentially introducing bias to pooled estimates. Similar to other reviews, we did not consider mortality amongst people lost to follow-up in our CFR estimates. Given that studies have suggested mortality among patients lost to follow up and transferred out averages 21% [57], our CFR is probably similarly underestimated [12]. As we did not consider studies focusing on special populations, our findings might not be generalizable to subpopulations with additional risk factors for mortality.

We found a high degree of heterogeneity between studies, which could be due to differences in clinical (e.g. CD4 count) or health system variables. We did not apply meta-regression because of the low number of studies, and the lack of information such as duration on ART and CD4 at start of TB treatment. By reporting results from random effects estimates, we acknowledge this heterogeneity. Due to the low number of studies contributing to the analysis, stratification by study quality was not possible. In addition, 95% of studies included in our systematic assessment have an observational study design which is subject to some risk of bias. However, since our aim was to describe the risk of death among HIV-infected TB patients under routine programmatic conditions, observational studies likely provide the most relevant data for this analysis.

In conclusion, we quantified the substantial impact of ART on reducing mortality during TB treatment. Collaborative tuberculosis-HIV activities are key components of the new Post-2015 Global Tuberculosis Strategy, approved by the World Health Assembly in May 2014 [58]. They include expanded collaboration between TB and HIV programmes and integrated tuberculosis and HIV service delivery in the field. For individuals newly diagnosed with TB, this means increased on-site HIV testing, and prompt referral for HIV care for all found to be HIV positive [59]. Improved harmonization relies on more sharing of clinical space and integration of medical records, staff, and training. These interventions promise to reduce delays to HIV diagnosis, facilitate early implementation of effective ART and reduce TB-related mortality in HIV positive patients.

## Supporting Information

Table S1 MEDLINE search strategy.
(DOCX)

Table S2 CD4 count at baseline and during follow up in the included studies.
(DOCX)

Table S3 TB treatment regimen in the included studies.
(DOCX)

Checklist S1 **PRISMA checklist.**
(DOC)
