# Supplementary material for: The Impact of Antiretroviral Therapy on Mortality in HIV Positive People during Tuberculosis Treatment: A Systematic Review and Meta-Analysis
Source: PLoS One. 2014 Nov 12;9(11):e112017. doi: 10.1371/journal.pone.0112017 (PMC4229142; doi:10.1371/journal.pone.0112017)
Supplement: Table S1 — MEDLINE search strategy. (DOCX) [file pone.0112017.s001.docx]

Table S1. MEDLINE search strategy

| **SET** | **MEDLINE** |
| --- | --- |
| 1  2 | Tuberculosis  TB |
| 3 | Sets 1-2 were combined with “OR” |
| 4  5  6 | Antiretroviral*  ART  HAART |
| 7 | Sets 4-6 were combined with “OR” |
| 8  9  10  11  12  13 | Case fatality  Mortality  Cause of death  Fatali*  Death*  Outcome* |
| 14 | Set 8-13 were combined with “OR” |
| 15 | Sets 3, 7 and 14 were combined with “AND” |
| 16 | Set 15 was limited to years “1996 to 2013” and English language |
