# Supplementary material for: The Impact of Antiretroviral Therapy on Mortality in HIV Positive People during Tuberculosis Treatment: A Systematic Review and Meta-Analysis
Source: PLoS One. 2014 Nov 12;9(11):e112017. doi: 10.1371/journal.pone.0112017 (PMC4229142; doi:10.1371/journal.pone.0112017)
Supplement: Table S2 — CD4 count at baseline and during follow up in the included studies. (DOCX) [file pone.0112017.s002.docx]

Table S2. CD4 count at baseline and during follow up in the included studies

| **Reference** | **Median CD4 count at the start of TB treatment [cells/mm^3^ (IQR/range)]** | **Median CD4 during follow-up**  **[cells/mm^3^ (IQR)]** |
| --- | --- | --- |
| Agodokpessi 2012 [[22](#_ENREF_22)] | - <200 in 121 (47%) subjects - 200-350 in 64 (25%) subjects - >350 in 49(19%) subjects - Unknown in 25 (9%) subjects   **<350 for 85 (33%) subjects included in the meta-analysis** | Na |
| Akksilp 2007 [[32](#_ENREF_32)] | Recorded in 195 (59%) subjects:  53 (1-873) for all subjects:   - <50 in 95(29%) subjects - 50-99 in 39 (12%) subjects - 100-199 in 36 (11%) subjects - >200 in 25 (8%) subjects   Unknown in 134 (41%) subjects  **Na for subjects included in the meta-analysis** | Na |
| Dean 2002 [[41](#_ENREF_41)] | 90 in 161 (86%) subjects divided in 3 treatment groups according to baseline median CD4 count:   - - 91 (25-220) CD4 for subjects not on ART - 65 (20-143) CD4 for subjects on dual ART - **59 (25-121) for subjects on HAART (n=47, 55% of subjects included in the meta-analysis)**   Unknown in 27(14%) subjects | at 6 months:   - (116-378 ) for subjects not on ART - (90-320) for subjects on dual ART - **(70-250) for subjects on HAART (n=42, 49% of subjects included in the meta-analysis)**   Data also available at 3, 9 and 12 month follow-up |
| Dos Santos 2013 [[39](#_ENREF_39)] | Na | Na |
| Ferroussier 2013 [[27](#_ENREF_27)] | Available for 900 (72%) subjects:   - 163 for all TB-HIV subjects - 113 in 90 subjects already on ART by the start of TB treatment **(19% of subjects included in the meta-analysis)** - 169 in 810 subjects not yet on ART   Unknown for 355 (28%) pts | Na |
| Gandhi 2012 [[14](#_ENREF_14)] | 78.5 (42-152) for all subjects (n=119):   - ≤50 in 40 (34%) pts - 51-200 in 63 (53%) pts - 200-350 in 16 (13%) pts   **Na for subjects included in the meta-analysis** | 211 after 12 months of ART |
| Henegar 2012 [[23](#_ENREF_23)] | Na | Na |
| Kaplan 2014 [[28](#_ENREF_28)] | Available for 37163 (95.3%) subjects:  161 (74-290) for all subjects |  |
| Kayigamba 2013 [[29](#_ENREF_29)] | Na | Na |
| Kendon 2012 [[24](#_ENREF_24)] | Whole cohort:   - ≤50 in 217 (47.7%) subjects - 51-100 in 117 (25.7%) subjects - 101-200 in 107 (23.5%) subjects - >200 in 14 (3.1%) subjects   Immediate ART (n=303, **78% of subjects included in the meta-analysis)**   - ≤50 in 181 (60.1%) subjects - 51-100 in 62 (20.6%) subjects - 101-200 in 46 (15.3%) subjects - >200 in 12 (4.0%) subjects   Early ART (**22% of subjects included in the meta-analysis)**   - ≤50 in 26 (30.6%) subjects - 51-100 in 31 (36.5%) subjects - 101-200 in 27 (31.8%) subjects - >200 in 1 (1.2%) subjects | Na |
| Nansera 2012 [[25](#_ENREF_25)] | On ART (data available on 68 subjects, **30%** **of subjects included in the meta-analysis):**  ≤100: 22 (32.4%) subjects  101-200: 20 (29.4%) subjects  >200: 26 (38.2%) subjects  Not on ART:  ≤100: 171 (54.3%) subjects  101-200: 63 (2%) subjects  >200: 26 81 (25.7%) subjects |  |
| Raizada 2009 [[33](#_ENREF_33)] | Na | Na |
| Sanguan­wongse 2008 [[34](#_ENREF_34)] | 54 (1-1169) for all pts (n=1269)  48 (1–1169) for pts on ART (data available for 624 subjects, **98%** **of subjects included in the meta-analysis)**  73 (1–1061) for pts not on HAART (n=643)   - <10 in 99 (8%) subjects (56 on ART, 43 not on ART) - 10-24 in 183 (14%) subjects (115 on ART, 68 not on ART) - 25-49 in 191 (15%) subjects (142 on ART, 49 not on ART) - 50-99 in 214 (17%) subjects (155 on ART, 59 not on ART) - 100-199 in 168 (13%) subjects (100 on ART, 68 not on ART) - ≥200 in 157 (12%) subjects (47 on ART, 110 not on ART)   Unknown in 257 (20%) subjects (11 on ART, 246 not on ART) | Na |
| Schmaltz 2009 [[40](#_ENREF_40)] | Available for 80 (75%) subjects:   - 169 (136-355) in 24 subjects on HAART >3 months - 125 (64-214) in 56 subjects not on ART or on HAART <3 months   Unknown in 26 (25%) subjects  **Na for subjects included in the meta-analysis** | Na |
| Sileshi 2013 [[30](#_ENREF_30)] | 114 (58-185) in 272 pts of on ART group (already on ART or started ART, **100%** **of subjects included in the meta-analysis)**   - 291 (183.5-448) in 150 pts of Non-ART group (did not receive ART) | Na |
| Sinha 2012 [[35](#_ENREF_35)] | 133 (7-588) in 88 pts on early HAART (2-4weeks), **59%** **of subjects included in the meta-analysis)**  152 (14-648 )in 62 pts on delayed HAART (8-12weeks), **41%** **of subjects included in the meta-analysis)** | Na |
| Tansuphasa­wadikul  2007 [[36](#_ENREF_36)] | 30 (1-97) (data available for 24 subjects, **29%** **of subjects included in the meta-analysis)** | Na |
| Tweya 2013 [[31](#_ENREF_31)] | Na | Na |
| Varma 2009 [[37](#_ENREF_37)] | Available for 653 subjects:   - 60 (23-146) for all subjects - 65 (28-166) among who survived - 30 (11-93) among who died   Unknown for14 subjects  **Na for subjects included in the meta-analysis** | Na |
| Zachariah 2007 [[26](#_ENREF_26)] | Na | Na |
| Zhao 2014 [[38](#_ENREF_38)] | 100 (40-208) for all subjects (n=768)   - 151 (50-243) in 191 subjects already on ART by the start of TB treatment **(45% of subjects included in the meta-analysis)** - 66 (28-142) in 238 subjects with early ART (within 90 days from TB treatment start) **(55% of subjects included in the meta-analysis)** - 119 (48-238) in 280 pts with delayed ART - 357 (125-505) in 59 pts who did not start ART | Na |
