# Supplementary material for: The Impact of Antiretroviral Therapy on Mortality in HIV Positive People during Tuberculosis Treatment: A Systematic Review and Meta-Analysis
Source: PLoS One. 2014 Nov 12;9(11):e112017. doi: 10.1371/journal.pone.0112017 (PMC4229142; doi:10.1371/journal.pone.0112017)
Supplement: Table S3 — TB treatment regimen in the included studies. (DOCX) [file pone.0112017.s003.docx]

Table S3. TB treatment regimen in the included studies

| **Reference** | **Type and duration of TB treatment** | **Details (if available)** |
| --- | --- | --- |
| Agodokpessi 2012 [[22](#_ENREF_22)] | Standardized: Benin national guidelines  *(Ministère de la santé, direction nationale de la protection sanitaire.* *Guide de surveillance épidemiologique et de prise en charge de la co-infection*  *tuberculose/VIH au Benin, 1re dition. Cotonou PNT, Septembre 2008; 56 p.)* | - New cases: (2HRZE/ 4HR) - Retreatment cases: (2RHZES/ERHZ/5ERH) |
| Akksilp 2007 [[32](#_ENREF_32)] | Standardized:  WHO guidelines  *(World Health Organization. Treatment of tuberculosis: guidelines*  *for national programmes. Geneva, Switzerland: The Organization;*  *2003. [cited 30 Apr 2007].Available from* [*http://whqlibdoc.who.int/hq/2003/WHO_CDS_TB_2003.313_eng.pdf*](http://whqlibdoc.who.int/hq/2003/WHO_CDS_TB_2003.313_eng.pdf) | - New cases: ‘patients received isoniazid, rifampin, ethambutol, and pyrazinamide’ |
| Dean 2002 [[41](#_ENREF_41)] |  | - 31% of patients: standard 6-month course - 69% of patients: prolonged anti-tuberculosis treatment (>6 months) |
| Dos Santos 2013 [[39](#_ENREF_39)] | Standardized: Brazilian national guidelines  *(Ministério da Saúde. Tuberculose: guia de vigilância epidemiológica. 1. Brasília, DF, Brazil: Fundação Nacional de Saúde, Ministério da Saúde; 2002. [Portuguese])* | - New cases: (2HRZ/ 4HR) - ‘Ethambutol was included for retreatment cases’ |
| Ferroussier 2013 [[27](#_ENREF_27)] | Na |  |
| Gandhi 2012 [[14](#_ENREF_14)] | Standardized: South African government-sponsored TB treatment program [(2HRZE) _5_ + (4HR)_5_]  *(Department of Health, Government of South Africa. The South African*  *Tuberculosis Control Programme Practical Guidelines. Republic of*  *South Africa Department of Health; 2000.* *Available at:* [*http://www.capegateway.gov.2a/eng/your_life/4502*](http://www.capegateway.gov.2a/eng/your_life/4502) *. Accessed October 23, 2008 )* | - [(2HRZEP) _5_ + (4HR)_5_] |
| Henegar 2012 [[23](#_ENREF_23)] | Standardized: DRC national guidelines  *(World Health Organization. Treatment of tuberculosis guidelines. 3rd ed. WHO/CDS/TB2003.313. Geneva, Switzerland: WHO, 2003)* | - New cases: (2HRZE/ 4HR) - Retreatment cases: extended 8-month regimen |
| Kaplan 2014 [[28](#_ENREF_28)] | Standardized: South African National TB management guidelines  *(World Health Organization. Consolidated guidelines on the use of anti-*  *retroviral drugs for treating and preventing HIV infection, 2013. Avail-*  *able at: www.who.int/hiv/pub/guidelines/arv2013)* | - New cases: (2HRZE/ 4HR) - Retreatment cases: [(2S)_5_ + (2HRZE) + (5HRE)] |
| Kayigamba 2013 [[29](#_ENREF_29)] | Standardized: Rwanda national recommendations (2HRZE + 4HR) | - New cases: (2HRZE/ 4HR) - Patients whose sputum smear has not converted after two months of treatment are prescribed an additional HRZE |
| Kendon 2012 [[24](#_ENREF_24)] | Na |  |
| Nansera 2012 [[25](#_ENREF_25)] | Standardized: Uganda national guidelines  *(Ministry of Health, Uganda. Manual of the National Tuberculosis*  *and Leprosy Programme. 2nd ed. Kampala, Uganda:*  *MoH, 2010.)* | - New cases: (2HRZE/ 6HE) - Retreatment cases: ‘treatment regimen does not comply with WHO recommendations and the practice is an operational approach as the country transitions to the recommended regimen’ |
| Raizada 2009 [[33](#_ENREF_33)] | Standardized: Indian national guidelines  *(Technical Operations Guidelines for Tuberculosis Control. (2005) New Delhi,*  *India: Central TB Division, Directorate General of Health Services, Ministry of*  *Health and Family Welfare, Government of India, http://www.tbcindia.org/*  *pdfs/Technical%20&%20Operational%20guidelines%20for%20TB%20Control.*  *pdf Accessed 1 May 2009.)* | - New cases: [(2HRZE)_3_ + (4HR)_3_] - Retreatment cases: [(2HRZES)_3_ +(HRZE)_3_ (6HRE)_3_] - Patients whose sputum smear has not converted after two months of treatment have their intensive phase   extended by one month |
| Sanguan­wongse 2008 [[34](#_ENREF_34)] | Standardized: WHO guidelines  *(Treatment of Tuberculosis: Guidelines for National Programmes. 3rd ed.*  *Geneva, Switzerland (WHO/CDS/TB/ 2003. 313): World Health*  *Organization; 2003. Available at: http://www.who.int/tb/publications/*  *cds_tb_2003_313/en/index.html. Accessed April 24, 2008.)* |  |
| Schmaltz 2009 [[40](#_ENREF_40)] | Standardized: Brazilian national recommendations (2HRZ + 4HR)  *(Castelo A, Kritski AL, Barreto AW, et al. II Consenso Brasileiro de Tuberculose. J. Bras Pneumol. 2004;30(Suppl 1):S24–S37)* | - (2HRZ + 4HR) - central nervous system TB: continuation phase extended to 7 months - Patients who developed severe hepatotoxicity: 3-month course of streptomycin, ethambutol, and oﬂoxacin followed by ethambutol and oﬂoxacin for 9 months. - Cases that received antituberculous regimens without rifampicin were treated for > 12 months. - Most cases of MDR: association of streptomycin (for 3 months), pyrazinamide (for 2 months), ethambutol, and ethionamide. - In cases treated with HAART regimens that precluded the use of rifampicin, this drug was replaced by streptomycin (for 3 months) plus ethambutol. |
| Sileshi 2013 [[30](#_ENREF_30)] | Na |  |
| Sinha 2012 [[35](#_ENREF_35)] | Standardized: Indian Revised National Tuberculosis  Control Programme (RNTCP) guidelines  *(Revised National Tuberculosis Control Programme (RNTCP): TB India 2010:*  *RNTCP Status Report. New Delhi: Ministry of Health and Family Welfare,*  *Government of India; 2010)*  *(Revised National Tuberculosis Control Programme (RNTCP): TB India 2010:*  *RNTCP Status Report. New Delhi: Ministry of Health and Family Welfare,*  *Government of India; 2010)* | - New cases: [(2HRZE)_3_ + (4HR)_3_] - Retreatment cases: [(2HR ZES)_3_ + (HRZE) _3_ + (5HRE) _3_] |
| Tansuphasa­wadikul  2007 [[36](#_ENREF_36)] | Na |  |
| Tweya 2013 [[31](#_ENREF_31)] | Standardized: WHO guidelines | - New cases: (2HRZE + 4HRE) |
| Varma 2009 [[37](#_ENREF_37)] | Na |  |
| Zachariah 2007 [[26](#_ENREF_26)] | Standardized: Malawi national guidelines  *(Ministry of Health and Population. Manual of the National*  Tuberculosis Control Programme of Malawi. 5th ed. Lilongwe,  Malawi: Ministry of *Health and Population, 2002.)*  *(World Health Organization. Treatment of tuberculosis: guide-*  *lines for national programmes. 3rd ed. WHO/CDS/TB/2003.*  *313. Geneva, Switzerland: WHO, 2003)* | - New cases: (2HRZE + 6HE) |
| Zhao 2014 [[38](#_ENREF_38)] | Na |  |
